# Global Transcriptome Profiles of 'Meyer' Zoysiagrass in Response to Cold Stress

**DOI:** 10.1371/journal.pone.0131153

**Published:** 2015-06-26

**Authors:** Shanjun Wei, Zhenlin Du, Fei Gao, Xiang Ke, Jing Li, Jianxiu Liu, Yijun Zhou

**Affiliations:** 1 College of Life & Environmental Sciences, Minzu University of China, Beijing, PR China; 2 Beijing Institute of Genomics, Chinese Academy of Sciences, Beijing, PR China; 3 Jiangsu Province and the Chinese Academy of Sciences, No.1 Qianhu Houcun, Zhongshanmenwai, Nanjing, Jiangsu Province, PR China; University of Western Sydney, AUSTRALIA

## Abstract

A long green period is essential for a turfgrass species with high ornamental value and a wide area of use. Zoysiagrasses (*Zoysia* spp. Willd.) are perennial turfgrass species popular in tropical, subtropical and temperate zones, possessing many properties necessary to be economically useful turfgrass. They do not have a long green period because of cold sensitivity. A main focus in zoysiagrass research is to develop cold tolerant cultivars. Understanding the cold response in zoysiagrass is a fundamental area of research. In the present study, ‘Meyer’ zoysiagrass (*Zoysia japonica*), a widely cultivated variety in the genus, is used. We employed RNA-Seq to investigate genome-wide gene expression profiles in leaves under cold stress (4°C). Using the Illumina sequencing platform, we obtained approximately 206 million high-quality paired-end reads from three libraries (0 h, 2 h, and 72 h cold treatment at 4°C). After de novo assembly and quantitative assessment, 46,412 unigenes were generated with an average length of 998 bp and an N50 of 1,522 bp. A total of 25,644 (55.2%) unigenes were annotated by alignment with public protein databases including NR, SwissProt, KEGG and KOG. Differentially expressed genes (DEGs) were investigated using the RPKM method. A total of 756 DEGs were identified between 0h and 2h-cold treatment, with 522 up-regulated and 234 down-regulated; and 5327 DEGs were identified between 0h and 72h-cold treatment, with 2453 up-regulated and 2874 down-regulated. The expression profile of 15 DEGs selected randomly was confirmed with qRT-PCR. The results suggest that cold stress can induce desiccation and oxidative stress, inhibit photosynthesis and substance transport. In response to the stress, genes involved in proline synthesis, in starch hydrolysis, in methionine and ascorbic acid metabolism, in SOD activity, and in DREBs response pathway were up-regulated. GA metabolism, ABA and JA stimulus response were affected under cold exposure. This is the first transcriptome sequencing of *Z*. *japonica*, providing a large set of sequence data as well as gene expression profiles under cold stress. It will improve our current understanding of the cold response of zoysiagrass and be beneficial in breeding research.

## Introduction

Zoysiagrasses (*Zoysia* spp. Willd.) are widely used, environmentally friendly warm-season perennial grass (Poaceae) species naturally distributed on seacoasts and grasslands around the Pacific. They spread by both rhizomes and stolons, and can tolerate wide variations in temperature, sunlight, and water. There are 16 species within the genus [1], most of which are sexually compatible [2]. Of these species, *Z*.*japonica* Steud is excellent at repelling weeds, holds up well under traffic, http://en.wikipedia.org/wiki/Zoysia-citenote-4resists disease, and requires little fertilization, so it is grown extensively for forage and for sustainable turf and golf courses in transitional and warm climatic regions throughout the world [3]. Lawns built up with zoysiagrasses can provide a higher quality playing surface, require fewer cultural inputs, have lower water and nutritional requirements, and possess good heat and drought tolerance, with generally good disease and insect resistance compared to other turfgrass species [4]. In transitional and temperate regions, however, the green period of zoysiagrass is largely limited by low temperature, which is the primary reason why it is not widely planted [5]. Breeding cultivars with cold tolerance and a long green period is one of the main focuses in zoysiagrass research. Understanding the cold response of this species at the molecular level should lead to the characterization of candidate genes important for genetic improvement.

Significant progress has been made in the past decade with model plants in elucidating transcriptional networks in the response to low temperature [6–7]. Generally, cold stress-inducible genes in plants can be classified into two groups. The first group consists of genes encoding proteins to protect cells from abiotic stresses, including genes for antioxidant enzymes, osmolyte biosynthesis, and molecular chaperones. The second group is made up mainly of genes that regulate stress signal transduction and gene expression, such as transcription factors and protein kinases. These findings are of use in understanding the cold response of zoysiagrass. Several studies have investigated cold-induced physiological changes, showing changes in the concentrations of sugars, organic acids, proline, soluble proteins and polar lipids under cold stress [8–10]. In a mapping group consisting of 96 zoysiagrass accessions, three SSR (simple sequence repeat) loci and one SRAP (sequence-related amplified polymorphism) locus were found to be associated with cold tolerance, and three SSR loci and two SRAP loci were found to be associated with the green period [11]. At the protein level, 70 protein spots were found differentially accumulated in stolons after a 14d-cold exposure [12]. A genome-wide investigation of the gene expression profiles would be helpful for our understanding of the cold response in plants as the gene regulation network is very complex [13].

Next-generation sequencing (NGS)-based RNA sequencing for transcriptome methods (RNA-seq) allows for transcript identification as well as potentially discovering genes involved in specific biological processes. This is especially suitable for non-model organisms whose genomic sequences are unknown [14]. In recent years, RNA-seq has emerged as a powerful method for discovering and identifying genes involved in abiotic stress resistance, dramatically improving our understanding of the interaction between plant and environment [15–16]. No previous genomic information has been reported in *Z*.*japonica*, and fewer than 500 ESTs have been deposited in Genbank for this species. In this study with cultivar ‘Meyer’, we used Illumina sequencing technology to determine the first comprehensive transcriptome for *Z*.*japonica*. By comparing the gene expression profiles of cold-treated and control zoysiagrass, we identified numerous differentially and specifically expressed transcripts of cold-regulated genes. These results will be a valuable resource for genetic and genomic studies for breeding cold tolerance of zoysiagrass.

## Material and Methods

### Plant material and cold treatment

‘Meyer’ zoysiagrass (*Zoysia japonica*) was collected from the nursery garden of turfgrass resources, Jiangsu Province and the Chinese Academy of Sciences. Dr Jianxiu Liu, one of the corresponding authors of this study, is in charge of the nursery garden and provided the plant material. This collection does not involve endangered or protected species. Grass patches of 10cm×10cm×10cm in size were grown in pots (20cm) in a greenhouse for 6 months, with a 14h light period and 20°C/25°C (Light/Dark) temperature conditions. For cold treatment, grass pots were transferred to a growth chamber set at 4°C for 2h (CT_2h) and 72h (CT_72h). Grass pots transferred to a chamber set at 25°C were used as the control (CK). The light period was 14h, and light intensity was 100 μmol m^-2^s^-1^. The second expanded leaf from the top was collected and stored at -80°C after freezing in liquid nitrogen.

### Physiological response assay

Relative water content [17], levels of total soluble carbohydrate, proline [8], and malondialdehyde (MDA) [18] were measured using previously described protocols. Activities of detoxification enzymes including superoxide dismutase (SOD), catalase (CAT) and peroxidase (POD) were determined according to Li &Yi (2012) [19]. One unit of SOD activity was defined as the amount of enzyme inhibiting NBT reduction by 50%. One unit of POD and CAT activity was defined as the amount of enzyme causing a change of 0.01 in absorbance per minute. Experiments were conducted with five biological replicates, and results were expressed as the mean ±standard error (SE). All data were subjected to one-way analysis of variance (ANOVA) and the LSD multiple comparison test (p < 0.05) using the SPSS statistical package.

### Total RNA extraction, RNA-seq library construction and sequencing

Total RNA was extracted using the Universal Plant Total RNA Extraction Kit RP3301 (Bio Take Corporation, Beijing, China) following the manufacturer’s instructions, with three biological replicates. RNA integrity was confirmed using an Agilent 2100 Bio Analyzer. Samples with RIN ≥ 7 and 28S:18S ratio ≥ 1.5:1, total amount 0.1∽4 ug, were considered acceptable. RNA samples from the same treatments were mixed and used for the determinations. Pair-end libraries were constructed as previously described [20] and sequenced on a HiSeq2000 system according to the manufacturer’s instructions (Capitalbio Company, Beijing, China). The raw sequence reads were deposited in the NCBI (National Center for Biotechnology Information) SRA database.

### RNA-seq data processing, de novo assembly and annotation

RNA-seq reads of each sample were first processed by removing the adapter sequence, then merged and assembled to transcripts using Trinity with default parameters (v.r2013-08-14) [21]. Alternative splicing transcripts were assigned to a unigene cluster. The longest transcript in a cluster was assigned as a unigene to form a set of non-redundant transcript sequences. Unigene sequences were aligned to a series of protein databases by BLAST (blastx, E-value < = 1e-5) (v2.2.6) following a priority order of Uniprot (Swiss-Prot/TrEMBL), Nr (non-redundant protein sequences in NCBI), KEGG (Kyoto Encyclopedia of Genes and Genomes database) and KOG (eukaryotic orthologous groups). The best hit of alignment was used to infer biological function of a unigene. The representative Gene Ontology annotation was performed by the software Blast2GO [22] which assigned homologous sequences aligned by BLAST with Uniprot and NCBI nr database to GO terms. We predicted transcription factors from protein sequences obtained by CDS predictions using hmmsearch to search the domains of plant transcription factors (http://plntfdb.bio.uni-potsdam.de/v3.0/). The transcription factor-related unigenes (E-value < = 1e-5) were classified according to the gene family information.

### Gene expression quantification and differential expression analysis

We used the Trinity platform for gene expression quantification and differential expression analysis, which included RSEM (v1.2.6) [23] for transcript abundance estimation, and normalization of expression values as FPKM (Fragments per kilobase of transcript permillion fragments mapped), and edgeR [24] for identifying differentially expressed genes (absolute value of log_2_ (ratio) ≥ 2and adjusted p-value < 0.001). GO terms and pathways enriched in the set of differentially expressed genes were calculated by the hypergeometrictest [25].

### Quantitative real-time PCR analysis

For quantitative real-time PCR (qRT-PCR) analysis of transcripts, 1 μg DNase I-treated total RNA was used to synthesize cDNA by M-MLV (Promega) using poly(dT) oligonucleotides. Actin-1 was used as an internal control. qRT-PCR was performed using One Step SYBR PrimeScript RT-PCR Kit (TAKARA, Dalian, China) according to the manufacturer’s instructions. Products were verified by melting curve analysis. Quantification was achieved by normalizing the number of target transcript copies to the reference Actin-1 gene using the comparative ΔΔCt method [26]. All reactions were performed with at least three biological replicates. Primers used in all quantitative RT-PCR experiments are listed in S1 Table.

## Results

### Physiological changes induced by low temperature

**Fig 1:**
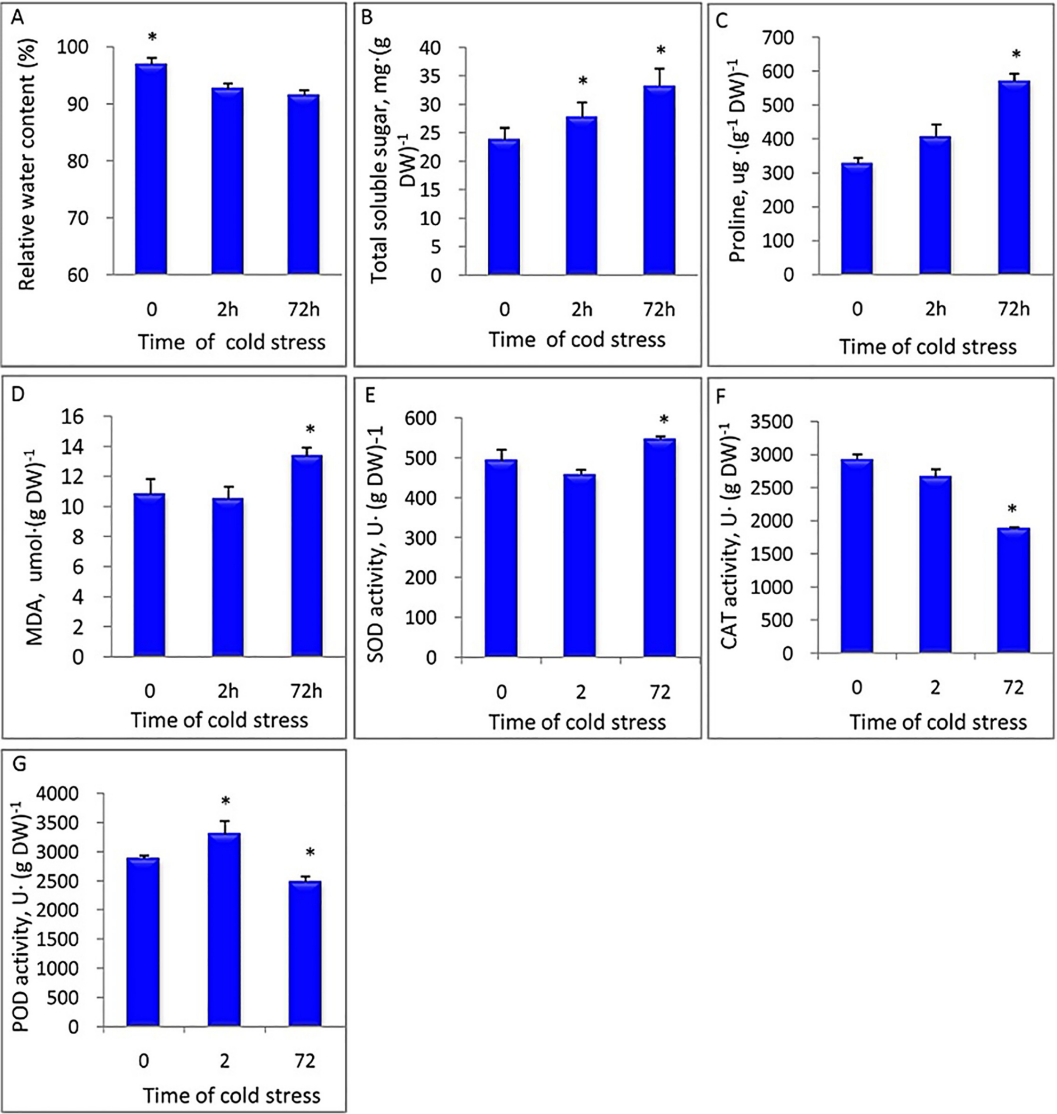
Physiological change in leaves under cold stress. ‘Meyer’ was placed in growth chambers at 4°C for the cold treatment and at 25°C for the control. The second expanded leaf from the top was used for determinations. Experiments were conducted with five biological replicates, and results were expressed as mean ± standard error (SE). *P < 0.05.

To investigate physiological responses in leaves, we measured relative water content (RWC) and levels of total soluble carbohydrate, proline and malondialdehyde (MDA). The activities of antioxidant enzymes, including SOD, CAT and POD, were also determined. After 2h at 4°C, RWC declined significantly, and the content of total soluble carbohydrate increased (P < 0.05). After 72h at 4°C, RWC remained constant, whereas soluble carbohydrate, proline and MDA increased (P < 0.05) considerably (Fig 1A–1D). The activities of SOD and CAT in leaves exposed to 2h did not change significantly (P ≥ 0.05), while the activity of POD increased by 14.67% (P < 0.05). After a 72h-cold exposure, SOD activity increased by 10.78%, while activities of CAT and POD decreased by 35.99% (P <0.05) and 13.85% (P <0.05), respectively (Fig 1E–1G).

### Illumina sequencing and reads assembly

To measure the transcriptome in the cold, we selected ‘Meyer’ leaves from normal condition (CK), 2h cold treatment (CT_2h), and 72h treatment (CT_72h) for RNA-Seq analysis. Three gene pair-end libraries, constructed from cDNA synthesized from leaf RNA, were sequenced with the Illumina HiSeq2000. Total clean nucleotides generated from each sample exceeded 5.5 Gb. We obtained 206,232,142 raw reads in total. Low-quality reads which contained adapter and unknown or low quality bases were discarded, and a total of 206,164,694 clean reads were obtained. Of these clean reads, the average Q30 percentage (sequencing error rate < 1%) was 88.47%. All clean reads were deposited in the NCBI and can be accessed in the Short Read Archive (SRA) under the study accession number SRP051412 (PRJNA270938).

**Fig 2:**
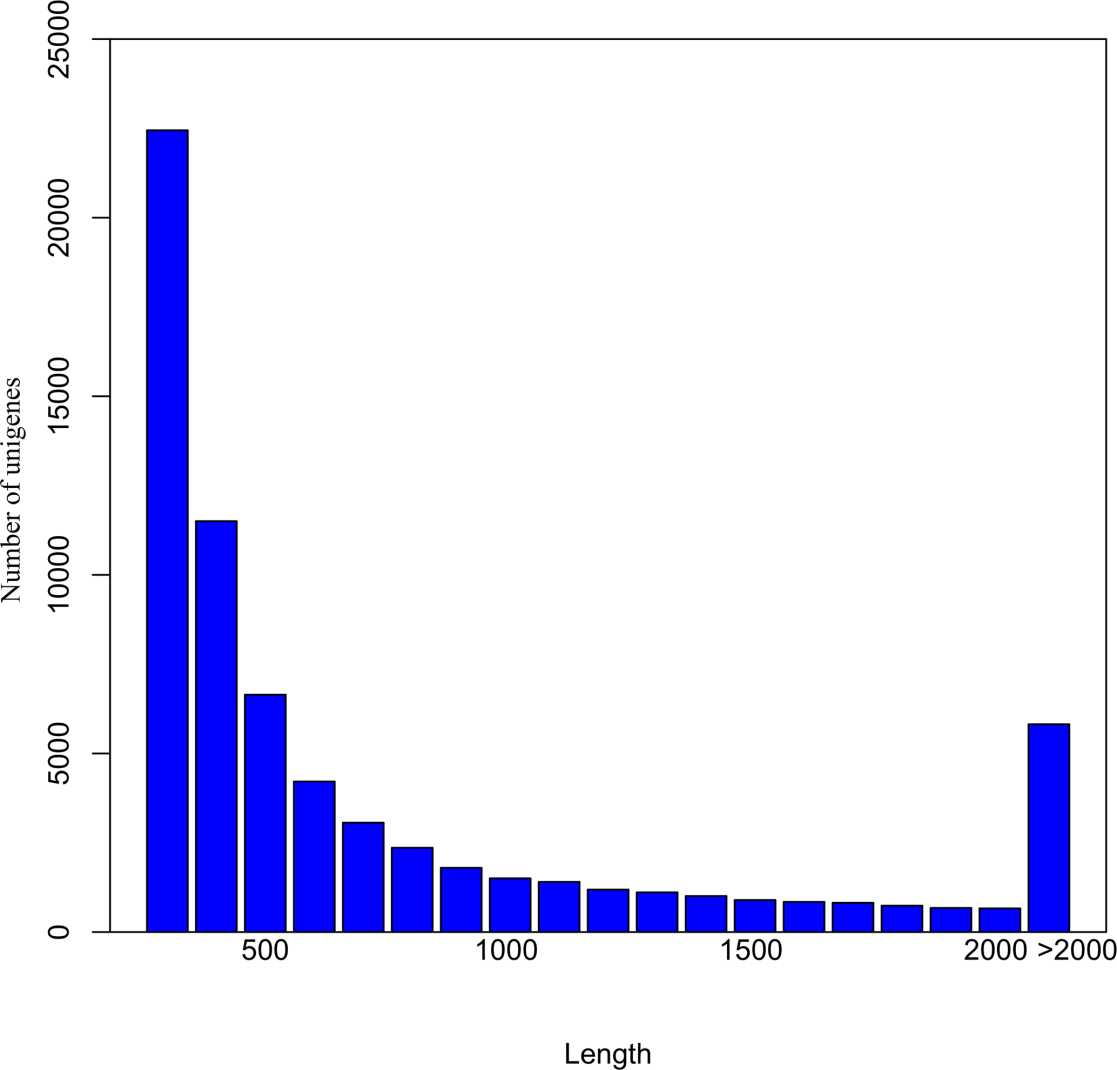
Length distributions of the unigenes. The X-axis lists the length of unigenes, and the Y-axis lists shows the number of unigenes of that size.

A total of 89,868 transcripts with length ≥ 300 nt were generated using Trinity software (Table 1). The mean transcript size was 1262 nt, and N50 is 1854 nt. Clustering analysis was used to assign all transcripts to 46,412 unigene clusters. A set of non-redundant unigenes was obtained by keeping the longest transcript in a cluster as representative. Among these unigenes, the mean length was 998 nt, with N50 being 1522 nt. A total of 28103 (60.55%) unigenes were longer than 500 nt, and 5818 (12.53%) unigenes were longer than 2000 nt (Fig 2), implying that a batch of transcripts with complete CDS were obtained. Reads were then mapped back to unigenes, 82.76%, 85.68%, and 83.60% of reads in libraries of CK, CT_2h, and CT_72h respectively, were mapped successfully, indicating that the transcript data was suitable for further analysis.

**Table 1:** Overview of the sequencing and assembly.

Total raw reads	206,232,142
Total clean reads	206,164,694
Q30 percent (%)	88.47
Transcript	
Total number	89,868
Total length (nt)	113,487,694
Mean length (nt)	1,262
N50 (nt)	1,854
Unigene	
Total number	46,421
Total length (nt)	46,341,662
Mean length (nt)	998
N50 (nt)	1,522

**Fig 3:**
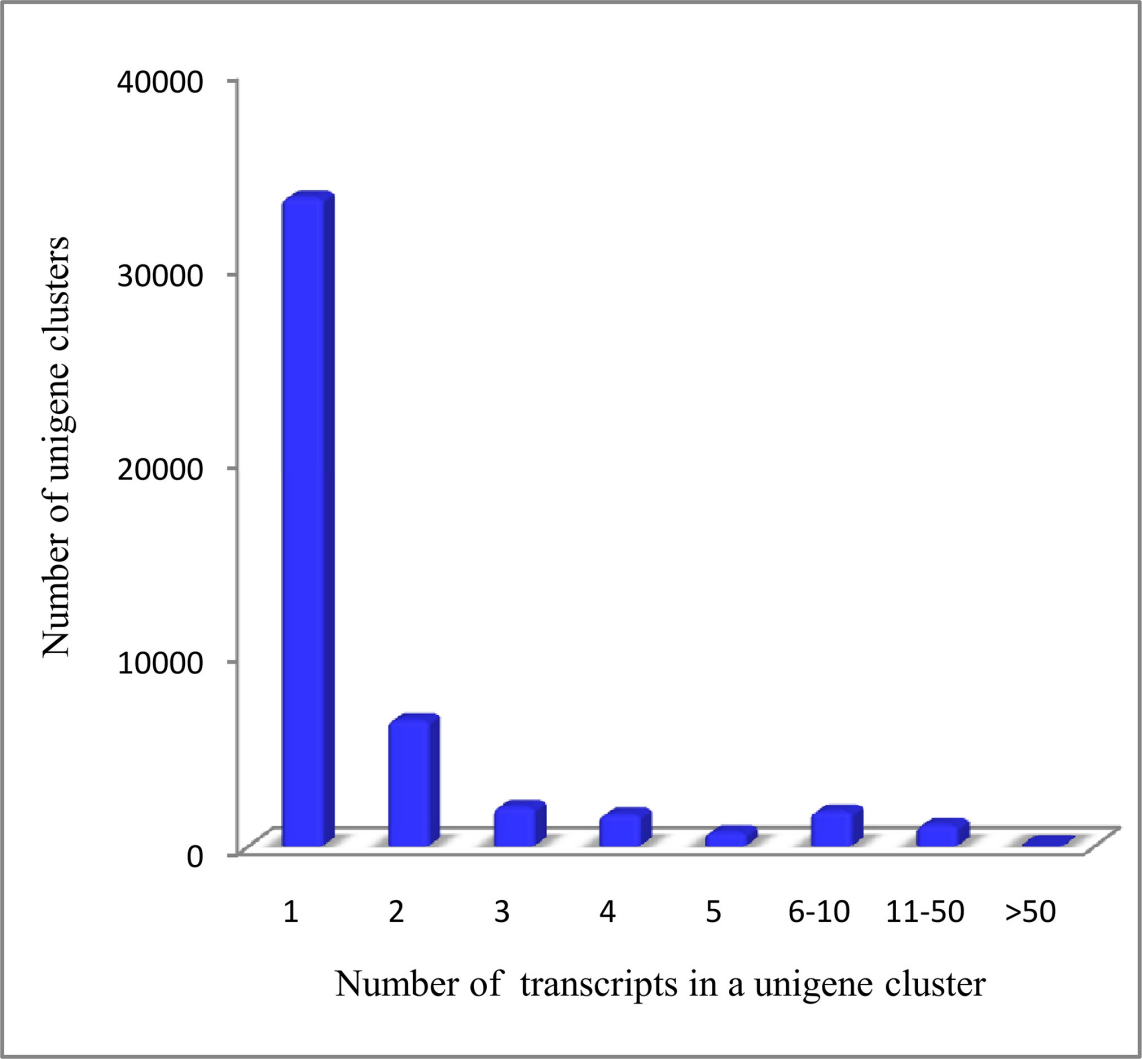
Histogram of Unigene clusters. The X-axis lists how many transcripts are in the unigene clusters, and the Y-axis lists show the number of unigenes clusters of that size.

Among the unigene clusters, 33,837 only contained one transcript sequence, while 12575 clusters contained more than one sequence (Fig 3). These multiple transcript unigene clusters can represent transcription variants, allelic variants, closely related paralogues, misassembled transcripts, or transcripts that were fragmented due to low coverage. The largest unigene cluster contains 188 transcript sequences and was blasted to a probable trehalose-phosphate phosphatase (TPP) gene family in *Setaria italic*. TPP removes the phosphate from trehalose-6-phosphate to produce free trehalose. There is also a large TPP gene family in the genome of rice and *Arabidopsis*, some of which are involved in the response to stress [27–28].

### Functional annotation and classification

**Fig 4:**
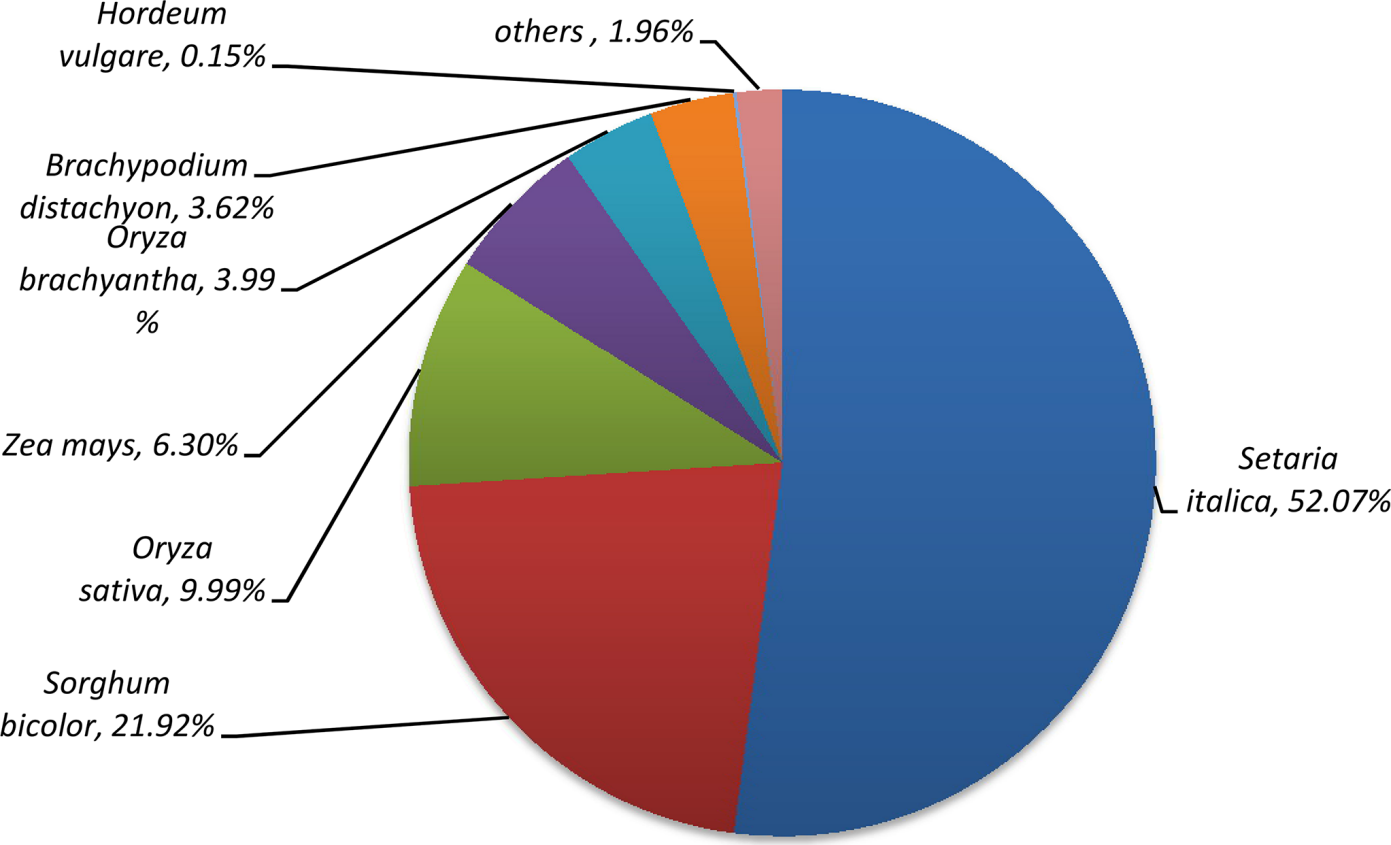
Species distribution of the BLAST hits.

We first annotated the unigenes by homologous searches against public databases in the priority order of Uniprot, Nr, KEGG and KOG. Using a cutoff value of E<10^−5^, a total of 25,644 (55.2%) unigenes had significant BLASTx hits in at least one of the four databases. Among them, 25438(54.8%), 25468(54.9%), 19031(43.1%), 13187(28.6%) unigenes were blasted to Uniprot, Nr, KEGG and KOG, respectively. A total of 12162 (26.2%) unigenes had hit in all four databases, while 20777(44.8%) unigene were not identified (Table 2). To study the sequence conservation of ‘Meyer’ in other plant species, we analyzed the species distribution of unigene datasets by aligning sequences against the Uniprot database. The species distributions for the best match from each sequence are shown in Fig 4. Specifically, 98.0% of these unigenes had the greatest number of hits to monocotyledonous species. The majority of unigenes had a significant level of sequence identity to foxtail millet (*Setariaitalica*) genes (52.07%). The second closest reference species was *Sorghum bicolor* (21.92%). *Oryza sativa* (9.99%), *Zea mays* (6.30%), *Oryza brachyantha* (3.99%), *Brachypodium distachyon* (3.62%), and *Hordeum vulgare* (0.15%) are reference species in decreasing order (Fig 4).

**Table 2:** Number of annotated unigenes (E < 10^−5^).

Database	Number	% of total unigenes
Total unigenes	46,421	100.0
Blasted total	25,642	55.2
Uniprot	25,438	54.8
Nr	25,468	54.9
KEGG	19,031	43.1
KOG	11,308	24.4
In all four databases	12,162	26.2
None annotated	20779	44.8

**Fig 5:**
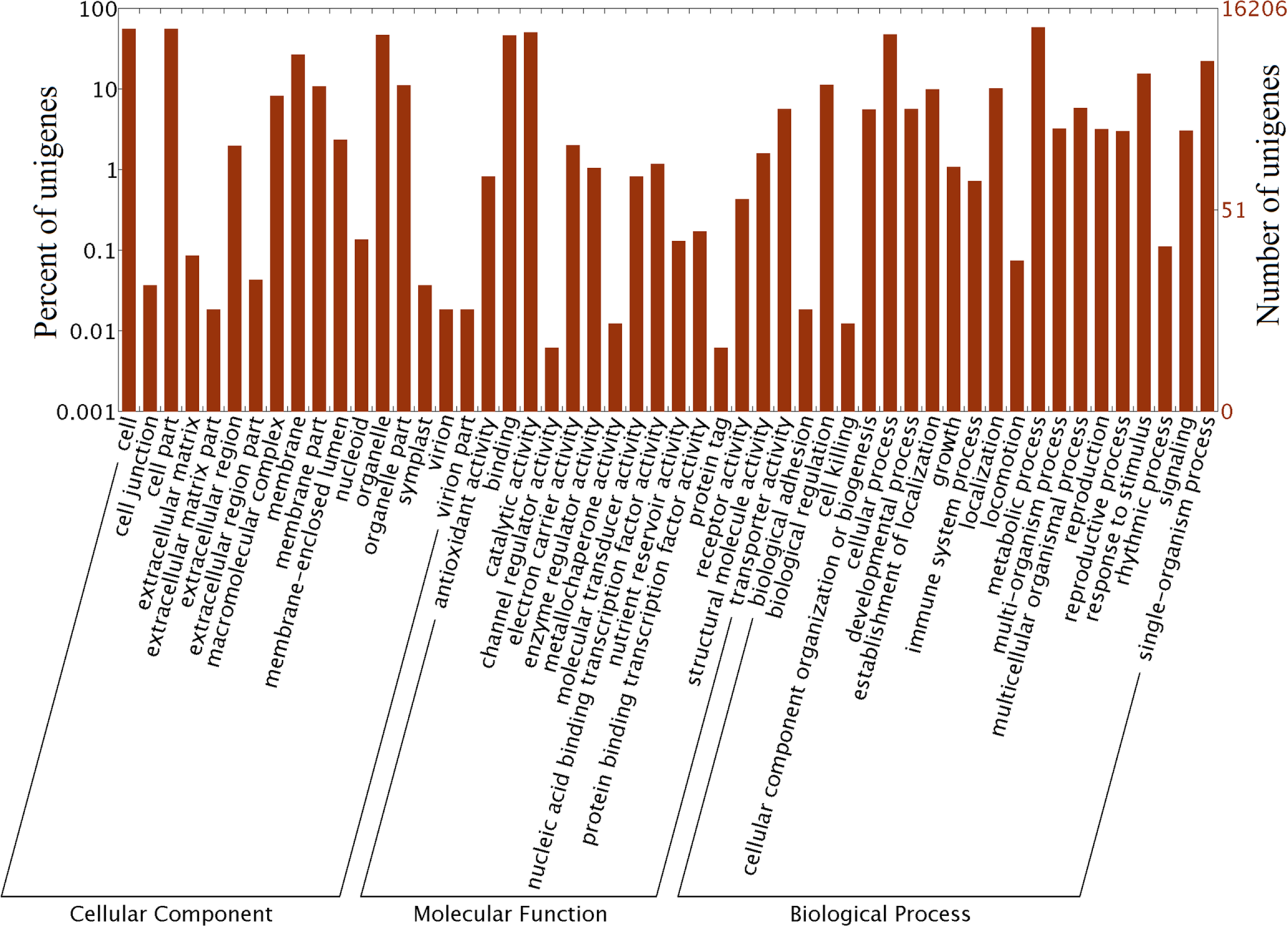
Gene Ontology (GO) classification of unigenes.

We used the Gene Ontology (GO) classification system to assess the possible functions of the unigenes. A total of 16,206 (16206/46421) unigenes were successfully assigned to at least one GO term (Fig 5). The unigenes were then classified into three categories: biological processes, cellular component, and molecular function. The category of biological processes consisted of 2021 GO terms, which were assigned to 82.94% of the unigenes; “metabolic process”, “cellular process” and “single-organism process” were the top three. The cellular component category consisted of 391 GO terms, which were assigned to 93.99% of the unigenes; “cell”, “cell part” and “organelle” were the major components. The category of molecular function consisted of 1359 GO terms, which were assigned to 73.66% of the unigenes; “catalytic activity”, “binding” and “transporter activity” were the top three functions.

**Fig 6:**
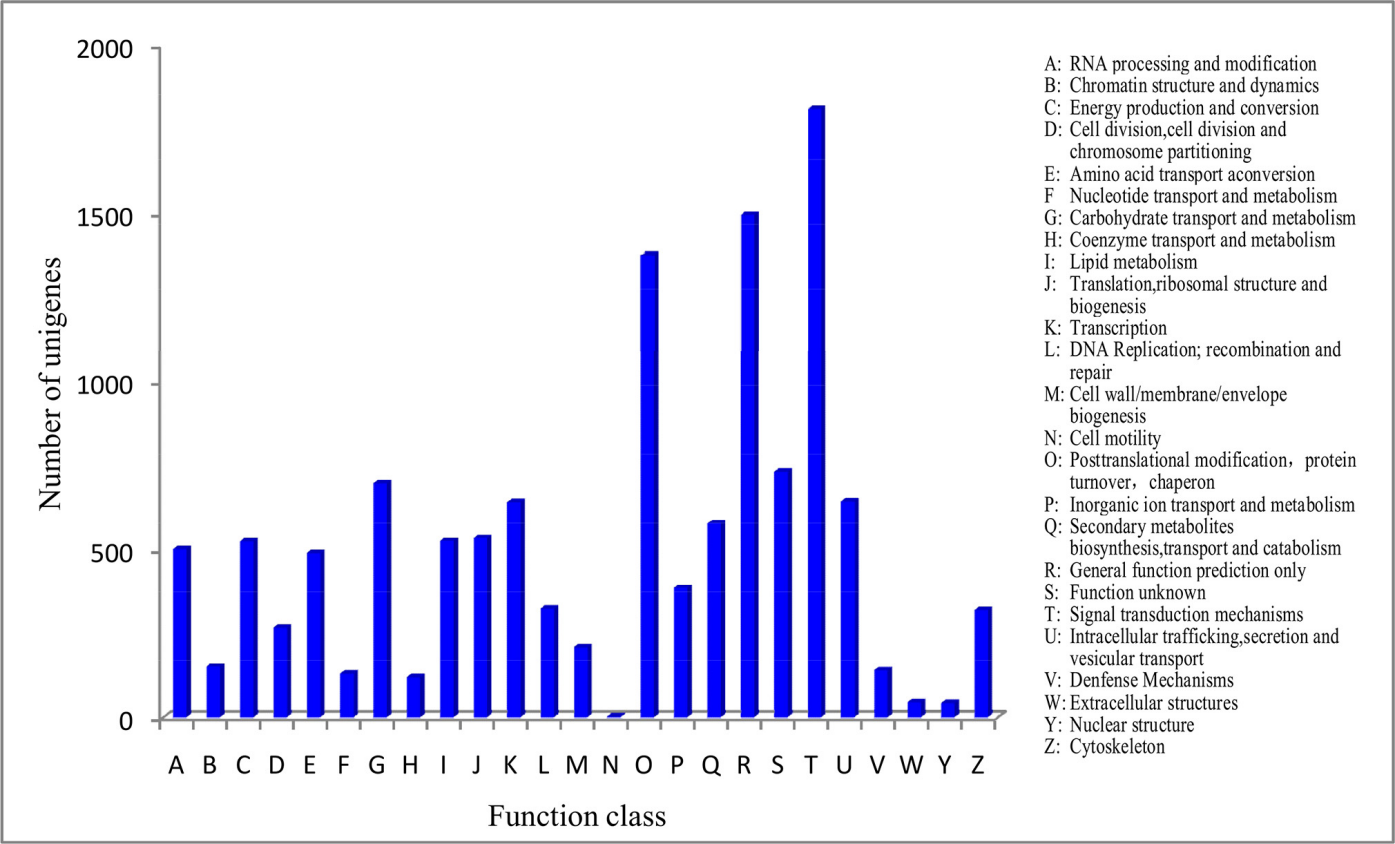
KOG function classification of unigenes.

We then aligned all the unigenes to the KOG database and 11,308 unigenes were identified. These unigenes were grouped into 25 functional categories (Fig 6). The top three categories were “signal transduction mechanism”, “general function prediction only”, and “posttranslational modification, protein turnover, chaperone”; the smallest category was “cell motility”.

Transcription factors are important upstream regulatory proteins and play critical roles in plant responses to abiotic stresses. In the present study we identified a total of 1979 transcription factors from the ‘Meyer’ unigenes that were classified into 55 different families (Table 3). The largest group of transcription factors was bHLH (203, 10.26%), followed by MYB-related family (144, 7.27%), ERF (132, 6.67%), NAC (130, 6.57%) and WRKY (127, 6.42%). These five families contributed up to 37.19% of the transcription factors identified in ‘Meyer’.

**Table 3:** Distribution of transcription factors by gene family.

Symbol	Number	Symbol	Number	Symbol	Number
bHLH	203	GeBP	30	E2F/DP	7
MYB_related	144	LBD	30	EIL	7
ERF	132	G2-like	28	ARR-B	6
NAC	130	Trihelix	27	CAMTA	5
WRKY	127	GATA	26	CPP	5
C2H2	97	Dof	21	DBB	5
bZIP	90	NF-YC	21	AP2	4
B3	77	TCP	21	LFY	4
M-type	76	HSF	18	SRS	3
S1Fa-like	74	ZF-HD	18	Whirly	3
FAR1	61	NF-YA	17	WOX	3
GRAS	61	SBP	17	GRF	2
C3H	59	Nin-like	13	HB-PHD	2
MYB	57	ARF	11	VOZ	2
YABBY	44	LSD	9	HRT-like	1
NF-YB	43	TALE	9	NF-X1	1
MIKC	41	BES1	8	RAV	1
HB-other	33	BBR-BPC	7		
HD-ZIP	31	CO-like	7	Total	1979

### Identification of differentially expressed unigenes

**Fig 7:**
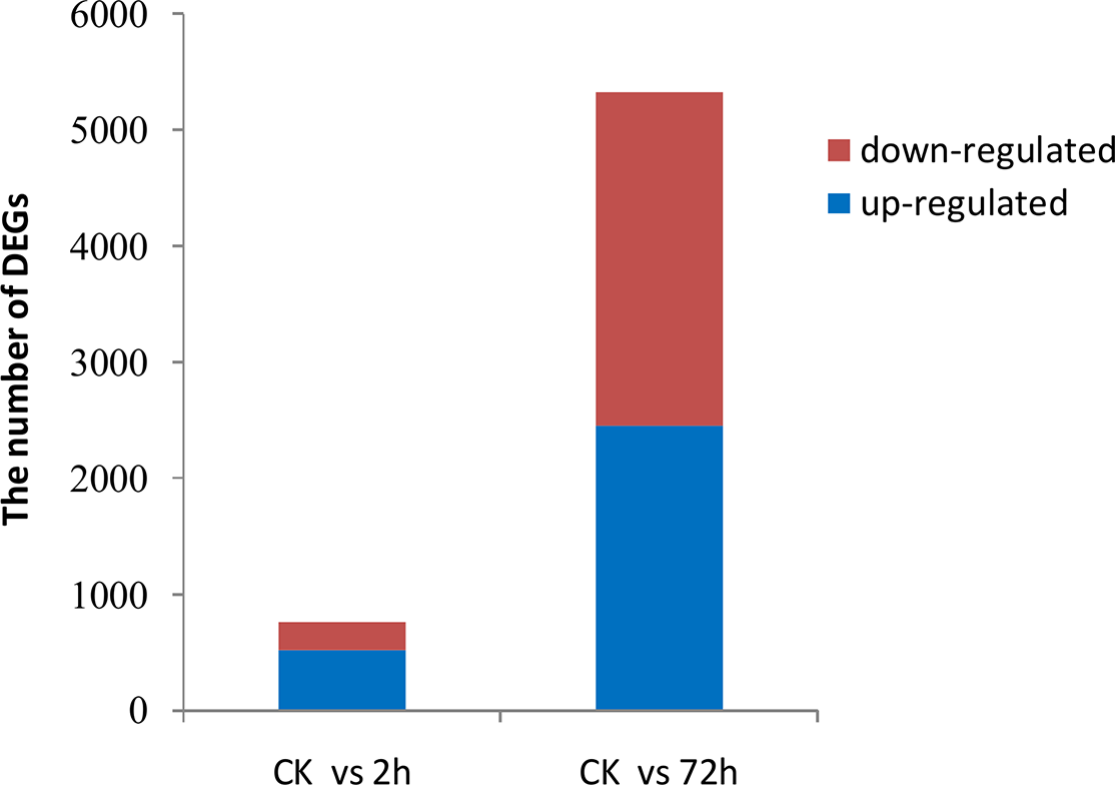
Number of DEGs between normal condition and cold treatments.

To identify a significant change in gene expression induced by low temperature, we used the FPKM method (Fragments per kilobase of transcript permillion fragments mapped) allowing us to calculate the expression levels of genes. Compared with CK, unigenes with absolute values of log_2_ (ratio) ≥ 2 and p-value ≤ 0.001 were considered as DEGs (differentially expressed genes). There were 756 DEGs between CK and CT_2h, including 522 up-regulated unigenes and 234 down-regulated ones (Fig 7, S2 Table). There were 5327 DEGs between CK and CT_72h, including 2453 up-regulated unigenes and 2874 down-regulated ones (Fig 7, S3 Table). To further validate our RNA-seq expression profile data, we performed qRT-PCR assays with primers designed according to fifteen randomly-selected unigenes (S1 Table). qRT-PCR data were normalized using the ‘housekeeping’ gene Actin-1.The results showed that gene expression profiles were consistent using either RNA-seq or qRT-PCR analysis, although the exact fold change varied between the two techniques (Table 4). The correlation coefficient between RNA-Seq data and qRT-PCR data is 0.837 (p < 0.01). In addition, PCR products of these primers showed the expected fragment size by agarose gel electrophoresis, which was consistent with the reliability of the assembled sequences.

**Table 4:** Verification of RNA-seq results by qRT-PCR.

unigene ID	Annotation (BLASTx)	Expression difference analysis of Illumina/Solexa (log_2_(CT_RPKM/CK_RPKM))		Relative gene expression by qRT-PCR (-ΔΔ CT)	
		2h/CK	72h/CK	2h/CK	72h/CK
comp36063_c0	Dehydration-responsive element-binding protein 1C	6.00	0.73	14.14±0.90	1.85±0.27
comp35206_c0	CBF3-like protein	5.43	-1.41	3.75±0.04	-1.22±0.17
comp33031_c0	CBF3-like protein	5.92	-0.35	4.50±0.04	-0.46±0.23
comp7425_c0	Dehydrin COR410	-0.86	6.96	-0.89±0.99	7.55±0.11
comp25704_c0	Carbohydrate transporter/ sugar porter	0.61	-12.77	-0.11±1.74	-6.25±1.8
comp23102_c1	Nitrate reductase	0.00	6.25	0.93±0.27	5.29±0.26
comp23102_c0	Nitrate reductase	NA^1)^	10.99	-0.22±0.99	4.48±0.71
comp16897_c1	Nitrate reductase	1.05	6.97	0.23±0.45	4.5±0.12
comp33067_c0	Hydrophobic LEA-like protein	NA	11.56	1.12±0.54	7.19±0.89
comp13382_c0	Stress-inducible membrane pore protein	NA	7.78	0.24±0.71	6.64±0.84
comp18155_c0	Vacuolar ATP synthase subunit D 1	NA	9.21	1.76±0.73	5.64±0.83
comp29027_c0	Vacuolar monosaccharide symporter 1	NA	6.48	0.64±0.41	3.46±0.17
comp31191_c0	Late embryogenesis abundant protein, group 3	NA	11.77	-0.42±0.18	9.85±0.10
comp38394_c0	Cis-zeatin O-glucosyltransferase 1	NA	8.55	0.58±0.05	5.79±0.33
comp5070_c0	Dehydrin;	NA	10.25	0.74±0.17	4.60±0.01

NA: not detected.

### GO and KEGG enrichment analysis of DEGs

To investigate the function of the DEGs, we then mapped all DEGs to the Gene Ontology database (http://www.genontology.org/) and calculated the gene numbers from each GO term. Using a hypergeometric test, we identified significantly enriched GO terms (p-value < 0.05) in DEGs compared to normal condition. After 2h at low temperature, 39 GO terms were enriched and can be categorized into 6 types (S4 Table): (1) Cell wall and external encapsulating structure organization; (2) Water and carbohydrate transport; (3) Electron carrier activity; (4) Tetrapyrrole binding; (5) Sequence-specific DNA binding transcription factor activity; (6) Auxin mediated signaling pathway. After a 72h-cold exposure, 112 GO terms were enriched (S5 Table). GO term types listed in (2) to (4) above were enriched more intensively. Other enriched GO terms in the CT_72h treatment were mainly related to functions such as: (1) Photosystem and photosynthesis activity; (2) Oxidation-reduction process; (3) Carbohydrate metabolism; (4) Nitrogenous compound metabolism and transport; (5) Lipid metabolism; (6) GA catabolism; (7) Response to ABA and JA stimulus.

KEGG enrichment analysis identified some biochemical pathways and signal transduction pathways. Compared with CK, 5 pathways showed significant change (p-value < 0.05) in the 2h-cold treatment, including biosynthesis of unsaturated fatty acids, biosynthesis of flavonoid and isoflavonoid, MAPK signaling pathway, and terpenoid backbone biosynthesis (S6 Table). In the 72h-cold treatment, 32 pathways showed significant changes (p-value < 0.05) (S7 Table). These pathways included: (1) Photosynthesis, including “antenna proteins”, “chlorophyll metabolism”, and “carbon fixation”; (2) Carbohydrate metabolism, including “starch and sucrose metabolism” and “pentose and glucuronate interconversions”; (3) Lipid metabolism, including “Linoleic acid metabolism” and “alpha-Linolenic acid metabolism”; (4) Antioxidants metabolism, including “ascorbate and aldarate metabolism” and “cysteine and methionine metabolism”; (5) Biosynthesis of secondary metabolites, including “phenylpropanoid biosynthesis”, “isoquinoline alkaloid biosynthesis”, and “flavonoid biosynthesis”; (6) Plant hormone signal transduction. The results of KEGG enrichment and GO term enrichment in the 72h-cold stress were in general similar.

Taken together, after exposure to 4°C for 2h, electron carrier activity and the biological processes of cell wall organization, water homeostasis and carbohydrate transport were affected. After 72 h of cold exposure electron carrier activity, redox balance, photosynthetic activity, carbohydrate and lipid metabolism, biosynthesis of some secondary metabolite, and organic substance transport were affected significantly. The metabolism of GA and responses to ABA and JA stimulus were also involved in cold stress response at the 72 h time point.

## Discussion

Temperature is an important environmental factor that affects the distribution, growth and development of plants. Cold stress, including chilling (<20°C) and/or freezing (<0°C) temperatures, prevents the expression of full genetic potential of plants owing to its direct inhibition of metabolic reactions and, indirectly, through cold-induced osmotic, oxidative and other stresses [6–7, 29]. To understand the cold response in ‘Meyer’ zoysiagrass at the molecular level, we compared the transcriptomes at normal growth temperature and at 4°C. The characteristics of the cold response in ‘Meyer’ are discussed below.

### Photosynthesis and nitrogen assimilation

Photosynthesis is greatly inhibited by low temperature in a number of plant species, of which the main impact is photosystem and its action [29]. Similar inhibition occurred in ‘Meyer’ under cold stress, indicated by the down-regulated expression of unigenes involved in porphyrin and chlorophyll synthesis, photosystem structure, photosynthetic electronic transport, and carbon dioxide fixation (S8 Table).

**Fig 8:**
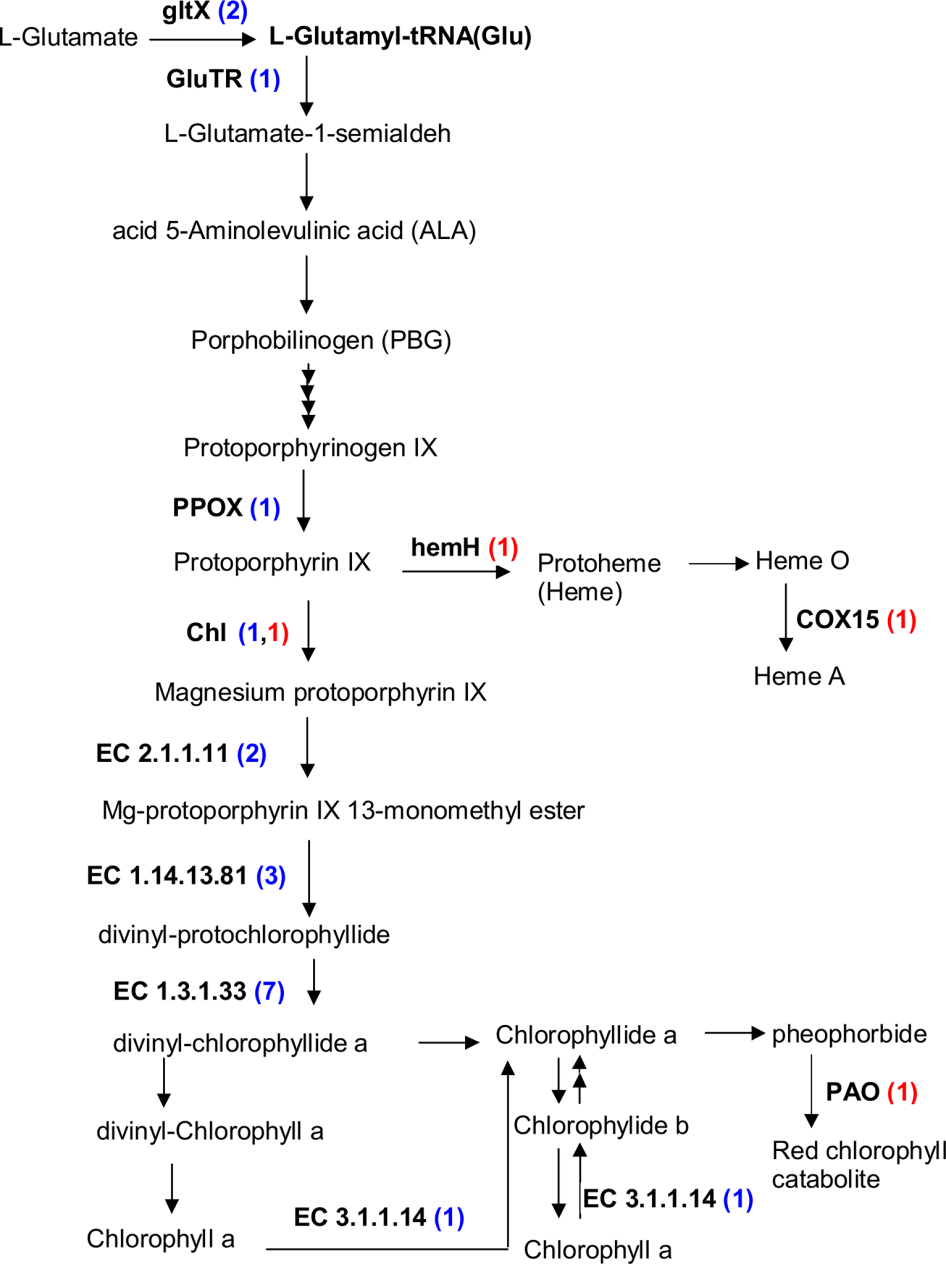
Chlorophyll metabolism in ‘Meyer’ under cold stress. gltX, glutamyl-tRNA synthetase; GluTR, Glutamyl-tRNA reductase; PPOX, protoporphyrinogen oxidase; Chl H, magnesium chelatase; EC 2.1.1.11, magnesium protoporphyrin IX methyltransferase; EC 1.14.13.81, magnesium-protoporphyrin IX monomethy lester (oxidative) cyclase; EC 1.3.1.33, protochlorophyllide reductase; EC 3.1.1.14, chlorophyllase; PAO, Pheophorbide a oxygenase; Hem H, ferrochelatase; COX15, cytochrome c oxidase 15 (Heme A synthase). In brackets, red numbers represent the number of up-regulated unigenes, blue numbers represent the number of down-regulated unigenes.

Chlorophylls are complex molecules exquisitely suited to the light absorption, energy transfer, and electron transfer functions carried out in photosynthesis. During the 72h-4°C stress, most of the DEGs involved in chlorophyll synthesis were down-regulated (Fig 8). At the same time, one unigene of ferrochelatase (Hem H), and one of pheophorbide a oxygenase (PAO) were up-regulated. Hem H catalyzes the insertion of ferrous iron into protoporphyrin IX to form protoheme, which departs from the chlorophyll biosynthetic pathway. PAO, the key enzyme in chlorophyll catabolism, catalyzes porphyrinmacrocycle cleavage of pheophorbide a (pheide a) to a primary fluorescent catabolite (pFCC) [30]; its expression was up-regulated during senescence [31]. Taken together, these gene expression changes suggest that chlorophyll biosynthesis decreases and chlorophyll catabolism increases during low temperature treatment.

An intact photosystem is necessary for effective light-absorption and energy transformation. In this study, 10 unigenes encoding chlorophyll a-b binding proteins, 8 unigenes encoding proteins in the reaction center of photosystem Ⅰ (PSⅠ), and 3 unigenes encoding oxygen evolving complex (PsbQ, PsbO and PsbR) were down-regulated. It can be inferred that light harvesting, the water-splitting reaction, and electron transport in PSⅠdecrease in leaves under low temperature exposure. The photosynthetic electron transport activity was also affected, as demonstrated by the down-regulated unigenes of plastocyanin and ferredoxins-1. These changes might lead to abnormal photosynthetic electron transport.

In the carbon fixation process, gene expressions of four enzymes, including carbonic anhydrases (CA), phosphoenolpyruvate carboxylase (PEPC), ribulosebisphosphate carboxylase (Rubisco) and fructose-1,6-bisphosphatase (FBP) were altered considerably during cold treatment. CA catalyzes the reaction of carbon dioxide with water to form bicarbonate, which is then transported to photosynthesizing cells. In this study, the expression of three unigenes encoding CA was down-regulated, suggesting that the exchange rate of carbon dioxide decrease during the 72h cold treatment. This result was consistent with previous research demonstrating that the exchange rate of carbon dioxide in zoysiagrass fell by 80–95% when canopy air temperature was 10°C-12°C, compared to the control (optimal temperature) [32]. PEPC, a key enzyme during C_4_ photosynthesis, has been suggested to be related to the cold sensitivity of C_4_ plants [33]. Findings in *Amaranthus hypochondriacus* (a NAD-ME type C4 species) have shown that PEPC activity fell to 70% on exposure to a low temperature of 15°C, but the protein content and phosphorylation of the enzyme did not change significantly [34] compared to the control. The present study showed that the expression of six PEPC unigenes changed significantly at 4°C, three were up-regulated and three were down-regulated. A previous study indicated that the activity of PEPC in zoysiagrass decreased drastically under 10°C/7°C (L/D) chilling stress [35]. The depressed enzyme activity might be related to the expression changes of PEPC isogenes under cold stress. In addition, one unigene of Rubisco small chain and one unigene of chloroplast located FBP were down-regulated. The expression changes of these carbon-fixation related unigenes support previous data showing that photosynthesis in zoysiagrass decreases greatly under chilling stress compared with that at 25°C [32].

In contrast to carbon fixation, nitrogen assimilation appears to be stimulated under cold stress. The expression changes of related unigenes are shown in S9 Table. First, the expressions of three nitrate reductase (NR) unigenes were up-regulated with fold changes of 6 to 11. Second, two unigenes, homologous with ferredoxin-6 and FNR root isozyme genes in *Zea mays*, respectively, were up-regulated. In *Zea mays* the two genes are nitrate-inducible and might function to transfer electrons from the NADPH to Fd-dependent enzymes such as nitrite reductase (NiR) and glutamine synthetase in root plastids [36–37]. Third, eight DEGs with GO term “glutamine family amino acid biosynthetic process” (GO:0009084), including two glutamine synthetase unigenes, were all up-regulated. NR catalyzes the reaction: NO_3_^–^+ NAD(P)H + H^+^+ 2 e^–^→NO_2_^–^+ NAD(P)^+^+ H_2_O. Nitrite can be converted to ammonia nitrogen by NiR, and the latter can then be assimilated as amide nitrogen through the glutamine synthetase pathway. It has been shown that in wheat seedlings the activity of glutamine synthetase and nitrate reductase increases during cold acclimation [38]. The overexpression of a glutamine synthetase gene in rice was found to enhance salt and chilling tolerance and increase photorespiration [39]. The up-regulated expression of these genes might increase nitrogen assimilation and photorespiration. It could be inferred that, owing to the decrease of carbon fixation under cold stress, nitrogen assimilation and photorespiration activities were enhanced so as to divert surplus assimilatory power—ATP and NADPH.

### Oxidative stress

Reactive oxygen species (ROS) are produced in plants as by products during many metabolic reactions, such as photosynthesis and respiration. Oxidative stress occurs when there is a serious imbalance between the production of ROS and antioxidant defense, causing rapid cell damage by triggering a chain reaction. In the present study, after a 72h-4°C exposure, a total of 270 DEGs were assigned to the GO term “oxidation-reduction process” (GO:0055114), including 177 down-regulated and 93 up-regulated with the fold changes of -10.54 to 13.16, compared to the control. The expression changes of these genes could affect some oxidation-reduction activities and trigger oxidative stress. GO enrichment analysis of DEGs indicated that GO terms “cellular response to reactive oxygen species” (GO:0034614) and “cellular response to oxidative stress” (GO:0034599) were enriched in the 72h-cold treatment. Oxidative stress can dramatically promote nonenzymatic lipid peroxidation [40], of which malondialdehyde (MDA) is one of the important end products. Physiological measurement showed that MDA levels in leaves increased significantly during the 72h treatment.

### Desiccation stress and inhibition in substance transport

Chilling stress can inhibit water uptake [41]. This study showed that the relative water content in leaves of ‘Meyer’ declined significantly (p < 0.05) after being exposed to 4°C for 2h, and remained at this level during the 72h exposure. Genes characterized by the GO term “response to desiccation” (GO:0009269) were enriched under cold stress for 2h and 72h, demonstrated by the down-regulated expression of two and four putative aquaporin unigenes, respectively (S10 Table). These results indicated that ‘Meyer’ became water stressed after cold treatment. Aquaporins are intrinsic membrane proteins that mediate the transport of water, small neutral solutes and CO_2_. It is well documented that regulation of aquaporins plays a role in early responses and acclimation to water stress [42]. In Arabidopsis, 12 aquaporin genes are down-regulated during cold acclimation and recover to original expression levels during de-acclimation [43]. The over-expression of aquaporin genes of *Rhododendron catawbiense* in Arabidopsis plants leads to a significant decrease in cold tolerance and the increase of dehydration rate of leaves [43]. The down-regulation of aquaporin unigenes thus might help to prevent water loss in ‘Meyer’ leaves under cold stress.

Desiccation stress might affect the activities of transporters. GO enrichment analysis of DEGs indicated that genes with GO terms “dicarboxylic acid transport” (GO:0006835) and “carbohydrate transport” (GO:0008643) were enriched during 2h-cold stress. After 72h-cold stress, genes with GO terms including “nucleobase transmembrane transporter activity” (GO:0015205), “nitrogen compound transport” (GO:0071705), “amino acid transmembrane transport” (GO:0003333), “peptide transport” (GO:0015833), and “carboxylic acid transport” (GO:0046942), were enriched. A total of49 DEGs, 37 of which were down-regulated, were found to have these GO terms, suggesting that transporter activities are inhibited during cold stress.

The affect of cold stress on “Carbohydrate transport” genes was the most noteworthy. After a 2h-cold exposure, three DEGs with this GO term, one encoding a bidirectional sugar transporter (SWEET1a) and two encoding monosaccharide transporter 3 (MST3) respectively, were up-regulated. SWEET1a mediates both low-affinity uptake and efflux of sugar across the plasma membrane. MST3 in rice is involved in the accumulation of monosaccharides required for cell wall synthesis during cell thickening [44]. It is possible that early sugar transport adjustment is involved in cell wall thickening, which is supported by the enrichment of DEGs with the GO term “cell wall organization or biogenesis” in the 2h-cold treatment. During the 72h-cold exposure, 14 DEG sassigned to the GO term “carbohydrate transport” were differentially regulated (S11 Table), including the nine down-regulated and the five up-regulated genes. Unigenes encoding sugar transporter proteins (STP), sucrose transport protein (SUT), hexose carrier, and polyol transporter were down-regulated. SUTs are responsible for the transport of sucrose into the cell with the concomitant uptake of protons (symport system) [45]. STPs have been shown to catalyze proton symport of hexoses across the plasma membrane into the cell, as demonstrated by heterologous expression in yeast [46]. The down-regulated expression of SUT and STP will restrict the phloem loading activity and, thus can affect the long distance transport of sugar. Unigenes encoding putative isoform plastidic glucose transporter 2, vacuolar monosaccharide symporter, and CMP-sialic acid transporter 3-like isoform X1 were up-regulated in the cold. In *Arabidopsis thaliana*, plastidic glucose transporter 4 is located in the chloroplast inner membrane and may be involved in the efflux of glucose into the cytosol [47]; the tonoplast monosaccharide transporter 1 has been shown to be involved in the transport of monosaccharides into the vacuole during cold stress [48]; and a CMP-sialic acid transporter is responsible for transporting CMP-sialic acid to the Golgi lumen [49]. It could be inferred that intracellular sugar transport was stimulated in ‘Meyer’ during the exposure to low temperature. Abnormal sugar transport in the cold could possibly lead to abnormal distribution of photosynthetic products in cells. Rogers et al [32] found that, when zoysiagrass was chilled at 10°C the chloroplasts accumulated starch and were substantially inflated.

### Resistance to desiccation stress

In response to drought stress brought about by low temperature treatment, osmotic adjustment occurs in ‘Meyer’, including the adjustment of potassium ion homeostasis and the accumulation of proline and soluble sugars. GO and KEGG enrichment analysis indicated that genes with the GO terms “potassium ion homeostasis” (GO:0055075), “proline biosynthetic process” (GO:0006561), and “starch and sucrose metabolic” KEGG pathway (ko00500) were enriched in ‘Meyer’ during the 72h-4°C stress.

Potassium (K^+^) is the most important and abundant cation in living plant cells and plays crucial roles in osmoregulation. In the enriched GO term “potassium ion homeostasis” two unigenes encoding calcineurin B-likes (CBLs) and ABC transporter C family member 5-like (abcC5-like) respectively were up-regulated, while a unigene encoding calcium-activated outward-rectifying potassium channel 1(KCa1) was down-regulated significantly (S10 Table). CBLs are commonly related to abiotic stress tolerance in plants [50], participating in the regulation of K^+^ uptake [51]; abcC5 is involved in regulation of K^+^ and Na^+^ cell content, and is required for regulation of stomatal opening by auxin, abscisic acid (ABA) and external Ca^2+^; KCa1, also known as two-pore potassium channel 1(TPK1), is apotassium-selective tonoplast ion channel functioning in K^+^ transport across the vacuolar membrane and plays a role in intracellular K^+^ homeostasis. The up-regulation of CBL and abcC5 accompanied with the down- regulation of KCa1 might change the distribution of K^+^ in apoplast, cytoplast and vacuolar to adapt to cold stress.

Proline is an important osmolyte in plant cells. It is also considered to be a scavenger of free radicals, an energy sink and a stress-related signal. The synthesis of proline is stimulated under multiple abiotic stresses [52]. In the present study, 6 DEGs with the GO term “proline biosynthetic process” (GO:0006561) were found to be up-regulated during the 72h-cold (S10 Table) but not during the 2h-cold exposure, which corresponded to the accumulation of proline in leaves.

Soluble sugars play an important role in osmotic adjustment. Sucrose and glucose accumulate and comprise the majority of the total soluble sugar in the stolon and rhizome of zoysiagrass during cold acclimation [8]. This study showed that the level of total soluble sugar went up significantly at the 72h-cold time point. At the transcriptional level, 102 DEGs were assigned to the “starch and sucrose metabolism” KEGG pathway (ko00500), including 34 up-regulated and 68 down-regulated. Among these, unigenes encoding starch branching enzyme (SBE), starch phosphorylase and α-amylase were up-regulated with fold changes of 3 to 12 in the 72h-cold treatment. SBE influence the structure of starch by catalyzing the formation of a-1,6-branch points with varied frequency and branch chain length. Overexpression of SBEIIb in an SBEIIb-deficient mutant of rice resulted in the accumulation of excessive branched, water-soluble polysaccharides instead of amylopectin [53]. The changes of these genes suggest that amylohydrolysis and starch-branching may contribute to the accumulation of soluble sugars in leaves. Sucrose synthase (SUS), a key enzyme involved in sucrose metabolism, catalyzes the reversible conversion of sucrose and UDP to UDP-glucose and fructose. Induced expression and increased activity of SUSs occurs in several species, such as Arabidopsis [54], wheat [55] and chickpea [56]. In the present study, three of the four SUS unigenes identified were induced with fold changes of 2.50, 2.35 and 1.29, respectively. During the reproductive stage of chickpea, a significantly higher specific activity of SUS was observed in seeds of most of the cold-tolerant lines but not in the cold susceptible lines [56]. It may be worthwhile to determine whether SUSs can be used as candidate genes to evaluate cold tolerance of different germplasm in *Zoysia* genus.

### Resistance to oxidative stress

Antioxidants play an important role in combating oxidative stress. Ascorbic acid (AsA) functions as a major redox buffer and as a cofactor for enzymes involved in regenerating other antioxidants. In the present study, genes with “ascorbate and aldarate metabolism” KEGG pathway were enriched at the 72h time point, in which two encoding GME (GDP-Mannose 3',5'-epimerase) were induced 2.3-fold and 6.2-fold, respectively. GME catalyzes an important step in the AsA biosynthetic pathway in higher plants. Overexpression of GMEs in tomato leads to AsA accumulation and enhanced tolerance to oxidative, cold, and salt stress [57]. The up-regulated expression of GME may enhance tolerance to oxidative stress in ‘Meyer’ exposed to low temperature.

Methionine (Met), a sulfur-containing amino acid, is highly susceptible to oxidation [58]. In arsenic-exposed rats methionine can result in the reversal of oxidative stress with a significant (P < 0.05) decline in tissue arsenic burden [59]. In the present study, genes with GO terms “Cysteine and methionine metabolism” KEGG pathway (ko00270) were enriched in ‘Meyer’ after a 72h-cold exposure, among which four encoding cobalamin-independent methionine synthase (MetE), homocysteine S-methyltransferase (HMT), S-adenosyl-l-methionine synthase (SAMS), and methionine γ-lyase (MGL) respectively, were up-regulated. Both MetE and HMT catalyze the synthesis of methionine, whose role in the stress response has been largely unreported. Methionine is converted to S-adenosylmethionine (SAM) which serves as a precursor for metabolites including glycinebetaine, methylated polyols, polyamines and ethylene. MGL is involved in cellular Met homeostasis and Ile synthesis. Transgenic Arabidopsis lines overexpressing a SAMS gene from potato exhibited higher salt and drought stress tolerance compared to controls [60]. MGL of Arabidopsis was up-regulated by simultaneous water deficit and nematode stress in root tissue, and conferred resistance to nematodes when overexpressed [61]. The up-regulation of the four genes could increase the level of Met and its derivatives, which may alleviate oxidative damage and enhance tolerance to cold in ‘Meyer’. The induced expression of MetE and HMT genes by low temperature provides a new insight into the cold response in plants.

Antioxidant enzymes such as superoxide dismutase (SOD), catalase (CAT) and peroxidase (POD) are important in reducing levels of ROS. Several of these enzymes have been shown to be induced under cold stress at either the transcript or protein level [62]. In the present study, the activity of POD increased during the 2h-cold stress, but declined significantly at the 72h-cold time point; the activity of SOD increased significantly during the 72h-cold stress; the activity of CAT declined during the cold stress. Compared with the control, two POD unigenes were transcriptionally up-regulated in the 2h-cold treatment, one SOD unigene was up-regulated but 12 POD unigenes were down-regulated in the 72h-cold treatment. These findings were consistent with the changes of POD and SOD activity. Three unigenes for CAT were identified and designated as comp35050_c4, comp30190_c0 and comp30190_c4. Under cold stress, the expression of comp35050_c4 increased, while that of the other two decreased. The decrease of CAT activity under cold stress suggests that the protein product of comp30190_c0 and comp30190_c4 could have great activity than that of comp35050_c4. The lower activities of CAT and POD under cold stress may be one of reasons why zoysiagrasses are cold sensitive. Increasing antioxidant enzymes activities should be considered in cold tolerance breeding research.

### Expression changes of DREBs and COR/dehydrin genes

The DREBs (Dehydration Responsive Element Binding proteins/C-repeat Binding Factors) proteins namely, DREB1 and DREB2, are thought to be the major transcription factors (TFs) that control stress-inducible gene expression in the ABA-independent pathway [63]. Overexpressing DREB genes in plants can significantly enhance plant tolerance to multiple abiotic stresses, including drought, freezing and high salinity [63]. Moreover, findings in some plants indicate that the different stress tolerance ability is related to diverse gene structures and expression patterns of DREB1 and DREB2. For example, in winter cultivars of wheat (*Tritic eaeaestivum* and *T*. *monococcum*) the constitutive and inducible expression of DREB1/CBFs within five pooideae-specific groups may play a predominant role in their superior freezing tolerance ability [64–65]; Among Arabidopsis natural populations, the relaxed selection on the CBF/DREB1s in some accessions from the southern part of Europe has compromised the ability of these genes to act as efficient transcriptional activators during the cold acclimation process [66]; In *Zea mays*, it was reported that natural variation in drought tolerance at the seedling stage was related to the variation of *ZmDREB*2.7 [67]. In this study, eight and five unigenes assigned to DREB1 and DREB2 subgroups, respectively, were identified (Table 5). Compared with the control, six DREB1 and two DREB2 unigenes were up-regulated significantly at the 2h-cold time point, and one DREB2 unigene was up-regulated at the 72h-cold time point. The induced expression of these DREB1 and DREB2 genes will be involved in increasing cold resistance in zoysiagrass. The relationship between the DREBs and cold tolerance in zoysiagrass is worthy of further research.

**Table 5:** Expression changes of unigenes of DREB1, DREB2 and COR/dehydrin.

Unigene	Annotation	logFC 2h Vs CK	logFC 72h Vs CK
comp30654_c0	Dehydration-responsive element-binding protein 1B	-2.826489	-2.54
comp32758_c0	CRT/DRE binding factor 1 [Chimonobambusa tumidissinoda]	-0.24	-2.10
comp32758_c1	CBF [Zoysia japonica]	2.12	-6.13
comp33031_c0	CBF3-like protein	5.92	0.35
comp33031_c1	CBF3-like protein	4.97	-1.31
comp33775_c0	dehydration-responsive element-binding protein 1A-like [Setaria italica]	5.06	-0.66
comp35206_c0	CBF3-like protein;	5.42	-1.41
comp36063_c0	Dehydration-responsive element-binding protein 1C	6.00	0.73
comp42390_c0	dehydration-responsive element-binding protein 2D-like [Setaria italica]	NA^1)^	8.06
comp9712_c0	DREB2A	-0.16	1.94
comp26632_c1	dehydration-responsive element-binding protein 2E-like [Setaria italica]	0.31	-1.41
comp35939_c0	DREB 2A;	2.01	0.89
comp29548_c0	DREB 2A	3.10	1.65
comp23961_c0	Cold acclimation WCOR413-like protein gamma form	0.12	-1.40
comp26778_c0	Cold acclimation protein COR413-PM1	-0.41	2.24
comp17770_c0	Cold acclimation protein COR413-TM1	-0.16	-2.09
comp14574_c0	Dehydration stress-induced protein	NA	5.19
comp24233_c0	Dehydrin	NA	5.87
comp25305_c0	Dehydrin	-0.08	0.77
comp29818_c0	Dehydrin	-0.11	1.03
comp5070_c0	Dehydrin	NA	10.25
comp7425_c0	Dehydrin COR410	-0.86	6.96
comp35700_c0	Dehydrin-/LEA group 2-like protein	0.52	6.79

NA: means not detected

A number of cold-regulated (COR) genes have been characterized in plants and some are identified as regulons of DREBs [7]. The dehydrins, belonging to the late-embryogenesis abundant (LEA) class of proteins [68], are among the COR genes that have been extensively studied [69] and shown to have major roles in conferring low temperature tolerance. Studies in wheat (*T*.*aestivum* L.) have shown that the expression of the COR genes Wcs120, Wcor410, and Wcor14 was highest for low temperature-tolerant and lowest for more cold sensitive genotypes [70]. In Arabidopsis, the expression levels of COR genes are higher in more cold-tolerant leaves than in cold-sensitive pollen [71]. In this study, ten unigenes annotated as dehydrin/COR were detected, six of which were up-regulated several fold during 72h-cold stress, indicating a potential relationship to the cold tolerance ability in ‘Meyer’.

The relationship of the COR413 family to cold tolerance is of interest. The COR413 family consists of two distinct groups: plasma membrane targeted (COR413-PM) and thylakoid membrane targeted (COR413-TM), respectively [72]. The expressions of one gene of group COR413-PM and one gene of group COR413-TM are closely associated with the acquisition of freezing tolerance in several plant species such as wheat, rye, and Arabidopsis. On the other hand, group *Cor413*-*pm* and-*tm* transcripts are not induced in the low temperature-sensitive species rice and maize [72]. In the present study, a unigene encoding COR413-pm 1 was induced by low temperature in ‘Meyer’, which is the reverse of the expression profiles in the low temperature-sensitive species mentioned above. Determining whether this induced expression is related to cold tolerance in zoysiagrass requires further research.

### Adjustment in phytohormone metabolism and signaling pathway

Phytohormones play important roles in the response of plants to stress. In this study, genes with GO terms “gibberellin metabolic process” (GO:0009685), “response to abscisic acid stimulus”(GO:0009737), and “response to jasmonic acid stimulus” (GO:0009753) were enriched after the 72h-cold exposure. The expression changes of DEGs in these GO terms are shown in S12 Table.

**Fig 9:**
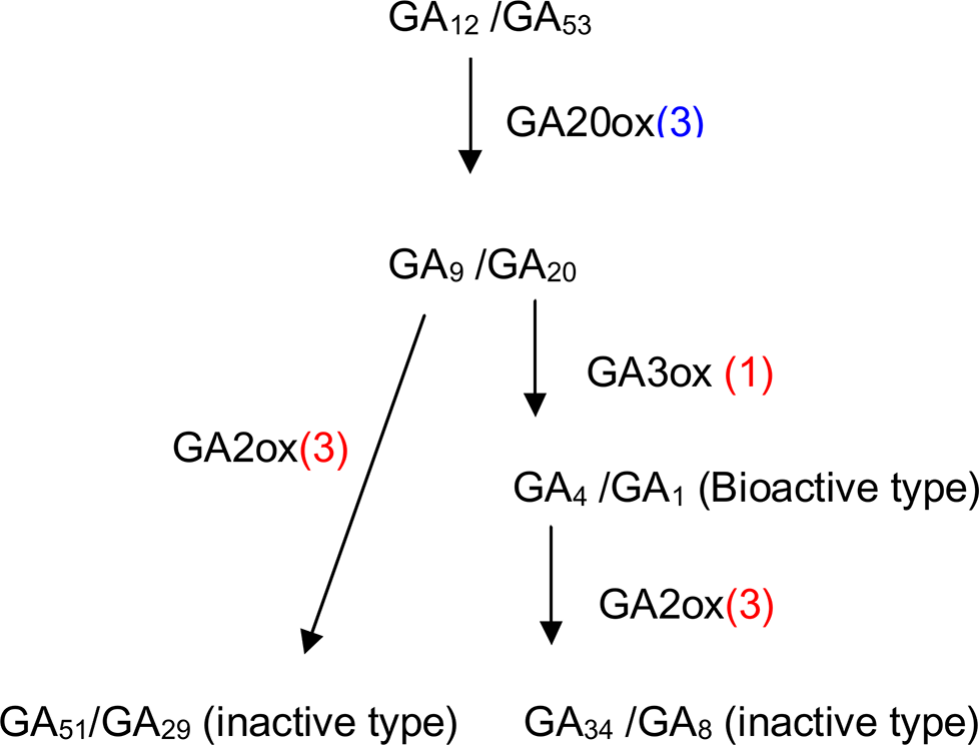
Gibberellin metabolism in ‘Meyer’ under cold stress. GA20ox, GA 20-oxidase; GA3ox, GA3-oxidase; GA2ox, GA 2-oxidase. Numbers in brackets represent numbers of unique transcripts regulated by cold stress. Red and blue indicate up- and down-regulated by low temperature, respectively.

Gibberellins (GAs) play a central role in plant development and the metabolism of GAs in plants is highly regulated [73]. From a regulatory standpoint, two biosynthetic enzymes—GA 20-oxidase (GA20ox) and GA 3-oxidase (GA3ox)—and an enzyme involved in gibberellin catabolism, GA 2-oxidase (GA2ox), are most notable. The gene transcription of these enzymes is highly regulated. In this study, three unigenes of GA20ox were down-regulated; three of GA2ox were up-regulated (Fig 9). These changes suggest that gibberellin catabolic pathways were activated and lead to the decreased endogenous level of bioactive GA just like other plant species [74].

ABA serves as an endogenous messenger in abiotic stress responses in plants, and has been called a ‘stress hormone’. Genes with the GO term of “response to abscisic acid stimulus” was enriched in ‘Meyer’ during the 72h-4°C exposure. Three regulators of ABA signal transduction pathways, including type 2C protein phosphatases (PP2C), sucrose nonfermenting 1-related protein kinase2 (SnRK2/ SAPK4), and proline-rich receptor-like protein kinase (PERK4), were up-regulated significantly in the cold. Without ABA, PP2C dephosphorylates SnRK2 kinases and blocks signal transduction. The ABA-bound receptor inactivates PP2C, which causes the SnRK2 kinases to be in a phosphorylated state, allowing them to activate bZIP group transcription factors (TFs) that then promote downstream ABA-responsive gene transcription [75–77]. PERK4, localized to the plasma membrane, has been shown to be an ABA- and Ca^2+^-activated protein kinase, is required for abscisic acid responses in Arabidopsis [78]. In this study, the expression of three PP2C unigenes, one SnRK2/ SAPK4 unigene and one PERK4 unigene were induced with fold changes of 2.53 to 7.10, suggesting that the ABA signaling pathway was stimulated in ‘Meyer’ exposed to cold.

Jasmonates, including jasmonic acid (JA) and its related metabolites, are essential components of the signaling pathway triggering the expression of plant defense genes in response to various sources of environmental stress [79]. Levels of endogenous jasmonates have been reported to increase upon pathogen infection, insect wounding, and various abiotic stresses [80–81]. In this study, genes with the GO term “response to jasmonic acid stimulus” (GO:0009753) was enriched during 72h cold stress, among which the regulation of allene oxide synthase (AOS) unigenes and indole-3-acetic acid-amidosynthetase GH3.12 unigenes were noteworthy. AOS is a major control point in *Arabidopsis thaliana* octadecanoid signaling. This enzyme acts on a number of unsaturated fatty-acid hydroperoxides forming the corresponding allene oxides. Several AOS enzymes have been cloned from various plant species. Most of them prefer 13-hydroperoxides as substrates and are involved in JA production [82–83]. JA can also induce the expression of AOS [84]. OsGH3.12 belongs to group I of GH3 genes in plants [85], which can conjugate JA with isoleucine to produce bioactive JA-Ile [86]. In ‘Meyer’ under 4°C stress for72h, two AOS genes were down-regulated with fold changes of -2 to -5, while AOS2-like, AOS3-like and AOS4-like were up-regulated 4 to 8-fold, and three OsGH3.12 homologs were down-regulated-7 to -9-fold. The expression changes of these unigenes suggest that metabolism and signaling pathways of JA are significantly regulated by cold stress in ‘Meyer’, which has also been reported in rice [81].

Cross-talk between the different plant hormones results in synergistic or antagonistic interactions that play crucial roles in the response to abiotic stress in many plants [87]

As shown in this study, the expression changes of multiple unigenes that are involved in metabolism and signaling pathway of different plant hormones might indicate a coordination of the regulatory network of stress signaling and cold response in zoysiagrass.

## Conclusion

In conclusion, this study provided the first large-scale transcriptome data set in warm-season turfgrass in response to low-temperature stress. More than 206 million high-quality 100-bp paired-end reads were generated and assembled into 46,412 unigenes in ‘Meyer’ zoysiagrass. Furthermore, 756 and 5327 unigenes were identified as DEGs in 2h- and 72h-4°C treatments, respectively; in addition, their classification, functions, and metabolic pathways are shown here for the first time. The down-regulated expression of genes involved in electron carrier activity, chlorophyll biosynthesis and carbon-fixation activity, water transport, organic substance transport, and antioxidant enzyme activity are consistent with the cold sensitivity of ‘Meyer’. The induced expression of genes involved in osmotic adjustment, antioxidants, nitrogen assimilation, phytohormone metabolism and signaling, and the DREB response pathway may play a vital role in cold resistance. The high-resolution expression patterns presented here further our understanding of the molecular response to low temperature in zoysiagrass, and would facilitate the breeding of cold tolerant varieties.

## Supporting Information

S1 Table Primers sequence for q-RT-PCR. (XLSX)

S2 Table The DEGs between 2h-cold stressand CK. (XLSX)

S3 Table The DEGs between 72h-cold stress and CK. (XLSX)

S4 Table The enriched GO terms under 2h-cold stress. (XLSX)

S5 Table The enriched GO terms under 72h-cold stress. (XLSX)

S6 Table The enriched KEGG pathwaysunder 2h-cold stress. (XLSX)

S7 Table The enriched KEGG pathways under 72h-cold stress. (XLSX)

S8 Table The expression changes of DEGs related to photosystem and photosynthesis. (XLSX)

S9 Table The expression changes of DEGs related to nitrate assimilation. (XLSX)

S10 Table The expression changes of DEGs involved in desiccation stress and osmotic adjustment. (XLSX)

S11 Table The expression changes of DEGs in GO term of carbohydrate transport. (XLSX)

S12 Table The expression changes of DEGs related to plant hormone metabolism or stimulus. (XLSX)
